# Host–gut microbiota interactions in health and disease: mechanisms and intervention strategies

**DOI:** 10.3389/fmicb.2026.1785607

**Published:** 2026-03-10

**Authors:** Yu Han, Zian Wang, Jiahao Xie, Guangdi Yang, Monong Su, Siqi Wang, Mengxin Yang, Huiyang Yu, Minghua Li, Liang Wang, Yunying Zhang, Binbin Hou

**Affiliations:** 1 Department of Dermatology, Second Affiliated Hospital of Dalian Medical University, Dalian, China; 2 Department of Gastrointestinal Surgery, Second Affiliated Hospital of Dalian Medical University, Dalian, China; 3 Laboratory Animal Centre, Dalian Medical University, Dalian, China; 4 Kunming Institute of Zoology, Chinese Academy of Sciences, Kunming, China

**Keywords:** bidirectional regulation, dysbiosis, gut microbiota, host, intervention strategies, mammals

## Abstract

The mammalian gut microbiota is a complex and dynamic “microbial organ” that interacts with its host. The gut microbiota contains a vast gene pool and metabolic capacity, producing key metabolites such as short-chain fatty acids (SCFAs), bile acids, vitamins, and other compounds. These metabolites regulate core physiological functions like energy metabolism, immune homeostasis, and neural behavior via the gut-brain axis (GBA), immune signaling networks, and other pathways. This review explores the bidirectional regulatory role of the gut microbiota. The gut microbiota influences the host’s metabolism and immune functions through its metabolites and structural components, while the host’s physiological state, internal environment, and lifestyle can alter the microbiota’s composition and function, creating a complex feedback network. Furthermore, the main mechanisms of dysbiosis in diseases are also explored. Dysregulation of the gut microbiota can damage the intestinal mucosal barrier, induce chronic inflammation, disrupt metabolic and immune signaling, and contribute to diseases such as type 2 diabetes, non-alcoholic fatty liver disease, inflammatory bowel disease, rheumatoid arthritis, and neurodegenerative disorders. Microbiota-based interventions, such as probiotics, prebiotics, and fecal microbiota transplantation (FMT), can be promising in disease management, but their clinical applications face challenges, including individual genetic backgrounds, lifestyles, and environmental factors, as well as difficulties in achieving long-term colonization of specific strains. Future research needs to uncover precise causal mechanisms in host-microbe interactions, as well as develop individualized microbiota intervention strategies to provide new theoretical bases and practical tools for the prevention, diagnosis, and treatment of major diseases.

## 1 Introduction

The gut microbiota of mammals is a complex and dynamic ecosystem composed of different microorganisms such as bacteria, viruses, and fungi. While recent studies suggest that the number of bacteria can be comparable to the number of host cells (Sender et al., 2016), genes in the microbiota genome outnumber the genes in the host genome by more than a hundredfold (Gilbert et al., 2018). This has led to the microbiota genome being referred to as the host’s second genome. The gut microbiota plays a vital role in metabolism, energy homeostasis, and immune function.

The gut microbiota, with its extensive metabolic capabilities, plays a role in the regulation of energy metabolism, immune homeostasis, and nervous system control. Since the idea of the “Gut-Brain Axis” (GBA) concept was introduced, it has become known that the gut microbiota and its metabolites communicate bidirectionally with the nervous system via neural, endocrine, and immune signals, affecting emotional behavior, cognitive function, and neural development (Socała et al., 2021). Importantly, the gut microbiota composition and function differ significantly between species, depending on long-term selective pressures from diet, evolution, and environmental adaptations. For example, herbivores generally have gut microbiota rich in bacteria that digest cellulose and hemicellulose, whereas carnivores have microbiota more adept at protein degradation (Milani et al., 2020). Furthermore, the host’s genetic background can affect microbiota composition, and the host genotype exhibits a degree of evolutionary conservatism regarding the microbiota’s ecological structure (Maritan et al., 2024).

The gut microbiota and the host have a long-term mutual relationship. The microbiota influences the host’s metabolic, immune, and neural functions via metabolites, microbial components, and signaling molecules. The host’s physiological state, diet, lifestyle, and immune environment affect the structure and function of the gut microbiota. When the structure and function of the gut microbiota undergo changes, such as a reduction in microbial diversity, a decrease in beneficial microbiota, or an increase in harmful microbiota, this phenomenon is commonly referred to as gut microbiota dysbiosis. Gut microbiota dysbiosis is, therefore, closely related to the onset and progression of various diseases. Broad changes in the composition, function, and metabolites of the gut microbiota are common in metabolic, immune-mediated, and neurological diseases (Ning et al., 2023; Chong et al., 2024; Son et al., 2025). Research suggests that dysbiosis may accelerate disease development by damaging the intestinal mucosal barrier, triggering chronic inflammation, and disrupting immune signaling pathways and metabolic homeostasis (Shen Y. et al., 2025).

Gut microbiota intervention techniques, including dietary regulation, probiotic and prebiotic supplementation, fecal microbiota transplantation (FMT), and specific drugs, have led to new approaches for the prevention and treatment of diseases. Many preclinical and clinical studies have shown that gut microbiota intervention techniques can be effective for metabolic, immune and neurodegenerative diseases (Zeng et al., 2022; Loh et al., 2024; Mederle et al., 2024). However, many challenges remain due to the high variability of microbiota composition among individuals and the limited stability of exogenous microbiota colonization in hosts. This is why the clinical application of gut microbiota intervention technologies urgently needs advances in mechanism analysis, personalized intervention program design, and translational studies.

This paper will systematically explore the core physiological functions and associated molecular mechanisms of the mammalian gut microbiota in nutrient metabolism, immune homeostasis, and neuromodulation. It will also analyze the bidirectional regulatory patterns of the gut microbiota in both healthy and diseased states. Following this, the paper will summarize the structural and functional changes in the gut microbiota, along with their pathological mechanisms, in metabolic, autoimmune, cardiovascular, and neurodegenerative diseases. Finally, it will review current intervention strategies targeting the gut microbiota, assess the development status of related research technologies, and analyze key issues in clinical translation.

## 2 The mutualistic symbiotic relationship between gut microbiota and the host

The gut microbiota of mammals, known as a “microbial organ,” produces a wide range of bioactive metabolites that play roles in the host’s multi-level physiological functions and contribute to maintaining homeostasis. This section explores mutualistic symbiotic interactions between the gut microbiota in host physiological functions, focusing on energy and material metabolism, immune regulation, and nervous system function.

### 2.1 Characteristics of the gut microbiota as a “microbial organ”

The mammalian gut microbiota is a dynamically interacting ecosystem within the host’s digestive tract. It contains various microorganisms, including bacteria, fungi, and viruses. The gut microbiota (sometimes referred to as a “microbial organ”) influences host evolution and physiological regulation through its wide range of genetic and metabolic capabilities. Among these microorganisms, bacteria are the most abundant and the most extensively studied. In humans, the gut contains approximately 10^13^ bacteria, making the colon one of the largest known microbial ecosystems (Sender et al., 2016). Metagenomic studies have identified more than 10 phyla of bacteria, but in healthy adults, Bacteroidetes and Firmicutes dominate the gut microbiota. This is due to the stable ecological balance achieved by long-term symbiosis between the host and its microorganisms.

Other microorganisms, such as viruses and fungi, are often overlooked, resulting in less research compared to bacteria and a lack of comprehensive understanding. However, with the advancement of multi-omics technologies, the limitations in understanding them are gradually diminishing. Gut fungi, which constitute about 0.1% of all gut microbes, play unique ecological roles, such as shaping microbiota structure, controlling host mucosal immunity, and regulating GBA signaling. Secondary metabolites produced by some gut fungi can be useful in treating infections and may have potential functional roles (Chin et al., 2020). The gut virome may rival or surpass that of bacteria, and its composition is strongly influenced by age, diet, and location (Cao et al., 2022). As viromics progresses, increasing evidence suggests that gut viruses may contribute to the maturation of the host immune system and possibly also play a role in inflammatory diseases, metabolic disorders, and many other complex conditions.

Genomically, the gut microbiota contains a gene count that surpasses the host genome by more than a hundredfold (Gilbert et al., 2018). The swift advancement of metagenome-assembled genomes (MAGs) technology has significantly broadened our comprehension of the genomic resources of uncultured microorganisms. The gene library contains various functional modules, such as fiber degradation, short-chain fatty acid (SCFA) production, and antibiotic resistance (Zou et al., 2019). These modules provide a molecular foundation for understanding the ecological role of bacteria, developing microbial intervention strategies, and elucidating the host’s disease mechanisms. They play a pivotal role in controlling the host’s health status due to their structure, function, and dynamic changes. Future work should improve the annotation of functional genes and verify mechanisms to understand how this community contributes to maintaining host homeostasis and disease progression.

### 2.2 Metabolic capacity and key metabolites of gut microbiota

The gut microbiota, with its extensive metabolic gene pool surpassing that of the host, can convert dietary substrates that are challenging for the host to digest into a wide variety of structurally diverse metabolites. These substances influence host metabolism and immune function locally or systemically while also affecting nervous system function via the GBA.

SCFAs, key products of gut microbial metabolism, are saturated fatty acids containing fewer than six carbon atoms. The gut microbiota contains numerous enzyme genes associated with carbohydrate activation and degradation. These enzymes break down dietary fiber and other fermentable carbon sources via metabolic pathways, such as the Wood-Ljungdahl and succinate pathways, primarily producing SCFAs such as acetate, propionate, and butyrate (Shen et al., 2021). When fiber intake is insufficient, more than 80% of gut microbes encode genes for the cleavage of mucin glycans, with some encoding genes for the catabolism of derived monosaccharides, suggesting that these monosaccharides can serve as alternative energy sources (Ravcheev and Thiele, 2017). SCFAs act as signaling molecules linking the microbiota to host physiological functions. They provide an energy source for colon cells and regulate various physiological processes, such as anti-inflammatory reactions, insulin secretion, energy homeostasis, liver metabolism, and brain function once they pass into the blood (Portincasa et al., 2022).

The biotransformation of bile acids (BAs) demonstrates how gut microbiota regulate host signaling molecules. Primary bile acids are synthesized in the liver from cholesterol. Once they enter the gut, they undergo conversion into secondary bile acids through enzymatic reactions, including bacterial deconjugation and dehydroxylation. Notably, 7α-dehydroxylation is a crucial step in the production of deoxycholic acid (DCA) and lithocholic acid (LCA) (Wise and Cummings, 2022). Beyond their role in lipid absorption and cholesterol metabolism, secondary bile acids act as endogenous ligands that activate host receptors such as the farnesoid X receptor (FXR) and G protein-coupled bile acid receptor (TGR5/GPBAR1) (Cai et al., 2022). This regulation of bile acid synthesis and transport enhances intestinal barrier function, maintains immune homeostasis, and strengthens host resistance to pathogenic bacteria (Wise and Cummings, 2022; Larabi et al., 2023).

The gut microbiota can synthesize various vitamins, providing the host with micronutrients in addition to those obtained from the diet. Although the absolute amount of microbiota-derived vitamins is often smaller than that of dietary vitamins, they are essential for energy metabolism, immune cell activation, and the regulation of inflammation (Yoshii et al., 2019). Vitamins are also important for the gut microbiota network, supporting the growth and metabolism of different microbes and maintaining community structure and ecological stability through competition and sharing (Tarracchini et al., 2024). These findings indicate complex cross-feeding relationships based on vitamin dependence within the gut microbiota, which are fundamental to its ecological functions as a “microbial organ.”

### 2.3 Regulation of host energy metabolic balance by gut microbiota

Mammals and their gut microbiota form a metabolic community that helps the body absorb nutrients and use energy efficiently. The gut microbiota has complementary enzyme systems and metabolic pathways, which help the host digest complex nutrients efficiently.

**FIGURE 1:**
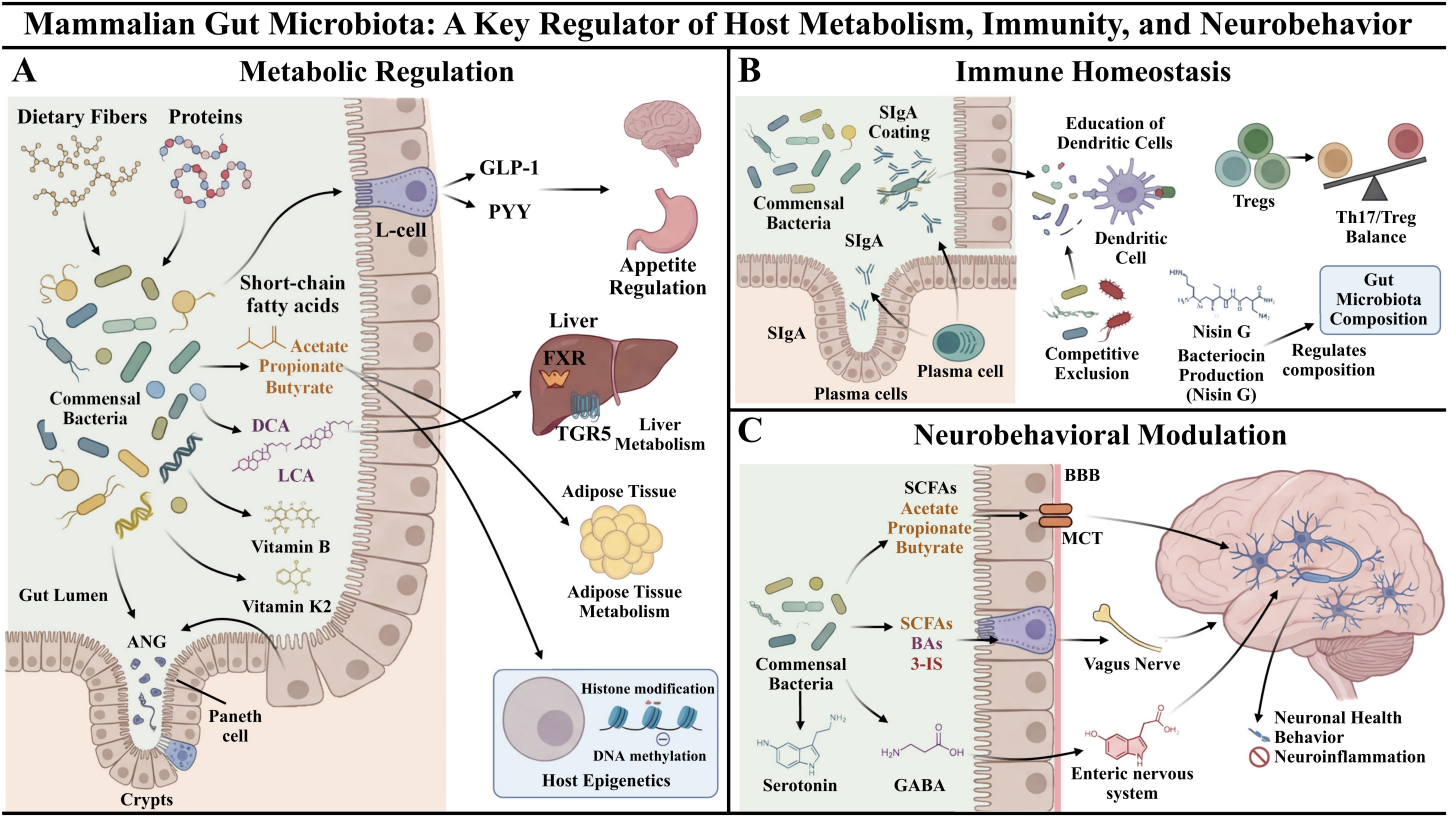
Mammalian gut microbiota. **(A)** Gut microbiota can regulate host metabolism by controlling hormone synthesis and secretion, communicating as signaling molecules, and changing epigenetic states. **(B)** Gut microbiota can maintain gut immune homeostasis by promoting host immune tolerance, competitively rejecting pathogenic bacteria, and secreting antimicrobial peptides. Host-derived antimicrobial peptides can also regulate the structure of bacteria in a reverse manner, forming bidirectional regulatory interactions. **(C)** Metabolites from gut microbiota can directly affect the central nervous system via the blood-brain barrier or indirectly activate the vagus nerve pathway to regulate nervous system excitability. Gut microbiota can synthesize various neurotransmitters that act on ganglion cells in the myenteric and submucosal plexuses of the enteric nervous system, transducing and integrating neural signals. GLP-1, Glucagon-Like Peptide-1; PYY, Peptide YY; TGR5, G protein-coupled bile acid receptor; PXR, Pregnane X Receptor; ANG, angiogenin; DCA, Deoxycholic Acid; LCA, Lithocholic Acid; SIgA, Secretory Immunoglobulin A; Th17, Helper T Cells 17; Tregs, Regulatory T Cells; SCFAs, short-chain fatty acids; BAs, bile acids; 3-IS, 3-indoxyl sulfate; MCTs, monocarboxylate transporters; GABA, γ-aminobutyric acid; BBB, blood brain barrier.

SCFAs, primarily produced by the gut microbiota through the fermentation of dietary fiber, play key roles in regulating energy metabolism. Butyrate is the main energy source for colonocytes and maintains intestinal barrier structure and function. Propionic acid enters the liver via the portal vein and can serve as a precursor to gluconeogenesis (Figure 1A; Mukhopadhya and Louis, 2025). While the gut microbiota composition influences the proportion of SCFAs, their production depends more on diet, providing a biological basis for metabolic regulation via diet (Arnoldini et al., 2025). SCFAs are also important metabolic signaling molecules that promote the release of glucagon-like peptide-1 (GLP-1) and peptide YY (PYY) from intestinal L cells (Mishra et al., 2020; Nazarinejad et al., 2025), suppress appetite, improve glucose metabolism, and enhance insulin sensitivity. SCFAs also mediate epigenetic regulation of host metabolic genes through DNA methylation, histone modification, and non-coding RNA regulation, improving obesity, chronic inflammation, and other metabolic diseases (Figure 1A; Kopczyńska and Kowalczyk, 2024).

Beyond carbon sources, the breakdown of proteins by the gut microbiota significantly affects host metabolism. The gut microbiota breaks down proteins into oligopeptides and amino acids. It produces metabolites like SCFAs, indole derivatives, and hydrogen sulfide (H_2_S) (Zhao et al., 2019). H_2_S acts in a dose-dependent manner. At physiological doses, it promotes immune homeostasis, reduces excessive inflammation, and aids tissue repair (Dilek et al., 2020). However, in cases of gut microbiota dysbiosis, especially with the overabundance of sulfate-reducing bacteria such as *Desulfovibrio*, H_2_S can inhibit GLP-1 secretion from L cells and disrupt glucose homeostasis and energy balance (Qi et al., 2024).

The gut microbiota regulates energy metabolism through bile acid metabolism. Secondary bile acids, such as DCA and LCA, are potent endogenous agonists of TGR5. TGR5 participates in energy, glucose, and lipid metabolism (Figure 1A; Wise and Cummings, 2022). During dysbiosis, however, the bile acid pool undergoes changes. For example, decreases in ω-muricholic acid (ω-MCA) and hyocholic acid (HCA) can reduce TGR5-mediated GLP-1 secretion, resulting in impaired blood glucose regulation and appetite control (Wang et al., 2023). Changes in the bile acid profile may play an important role in microbiota-related metabolic diseases.

Vitamins produced by the gut microbiota are important for the host’s energy metabolism. B group vitamins, key components of coenzymes for various metabolic enzymes, are involved in energy processes such as the tricarboxylic acid cycle, fat oxidation, and the electron transport chain (Yoshii et al., 2019). Vitamin K_2_ (e.g., menaquinone-4) is a ligand for pregnane X receptor (PXR) and regulates the expression of genes related to hepatic bile acid synthesis and energy metabolism (Sultana et al., 2018). Vitamin B9 supplementation can change the microbiota structure, regulate lipid metabolism and energy intake, and boost the expression of metabolic genes (Chen S. et al., 2022). This suggests that vitamins, the microbiota, and the host have a network of synergistic regulation.

In summary, the gut microbiota enhances the metabolic capacity of the host genome through complex metabolic interactions and is crucial for maintaining energy balance. Dysbiosis can disrupt hormonal and metabolic signals, reprogram tissue energy metabolism, and trigger chronic, low-grade inflammation (Zarrinpar et al., 2018).

### 2.4 Maintenance of immune system homeostasis by gut microbiota

#### 2.4.1 The role of gut microbiota in the establishment of host immune tolerance

Gut bacteria must also establish and maintain effective immune tolerance within the host to avoid being eliminated by the immune system. Antigens from gut bacteria are collected by microfold cells in Peyer’s patches (PPs) and isolated lymphoid follicles (ILFs). These cells initiate the humoral immune response of the mucosa-associated lymphoid tissue. This reaction stimulates B cells to differentiate into IgA-producing plasma cells that produce secretory immunoglobulin A (SIgA). SIgA encapsulates or confines bacteria in a non-inflammatory manner, preventing excessive adhesion to epithelium or transmucosal translocation (Figure 1B; Pietrzak et al., 2020).

The intestinal cells produce specific proteins that target and bind to commensal bacteria, which produce outer membrane vesicles (OMVs). The structures and signaling molecules of OMVs enhance the intestinal mucosal barrier and support local immune homeostasis (Yang et al., 2025). The role of OMVs in host control of the microbiota highlights them as potential immune adjuvants.

The gut microbiota affects pattern recognition receptors (PRRs) on the surface of intestinal epithelial or immune cells to regulate the differentiation of T cell subsets and immune tolerance. Helper T cells (Th) drive pro-inflammatory reactions to fight infection, and regulatory T cells (Tregs) maintain immune homeostasis by inhibiting excessive immune responses. Hence, the balance between Th cells and Tregs is necessary for immune tolerance. Research shows that cell surface β-glucan/galactan (CSGG) on *Bifidobacterium bifidum* partially interacts with Toll-like receptor 2 (TLR2) on dendritic cells (DCs), enabling DCs to adopt an immunosuppressive phenotype and secrete anti-inflammatory mediators, leading to differentiation of Treg cells and eventually immune tolerance (Figure 1B; Verma et al., 2018).

Furthermore, the gut microbiota-derived amino acid L-lysine is taken up by DCs and activates the aryl hydrocarbon receptor (AhR) and the indoleamine 2,3-dioxygenase 1 (IDO1)-kynurenine metabolic pathway. This activity increases the differentiation of naïve CD4^+^ T cells into Tregs and controls the Th17/Treg balance (Zhang et al., 2024).

In conclusion, certain probiotics and their metabolites are vital for promoting immune tolerance and maintaining mucosal immune balance. This understanding offers a novel theoretical foundation and potential therapeutic strategies for addressing inflammatory bowel disease (IBD), food allergies, and other immune-related conditions.

#### 2.4.2 Gut microbiota and defense against pathogens

The gut microbiota and intestinal epithelial cells act as the first line of defense against intestinal pathogen invasion. The intestinal epithelial cells form a physical barrier through structures like tight junctions, which can be regulated by the gut microbiota. For instance, probiotics can increase the expression of tight junction proteins, which decreases permeability and the risk of pathogen trans-epithelial migration (Du et al., 2025). In addition, the gut microbiota stimulates goblet cells to secrete mucins, which aid in mucus formation and prevent pathogens from swarming to the epithelial surface (Bergstrom et al., 2020).

In addition to reinforcing the physical barrier, the gut microbiota prevents pathogenic bacteria from colonizing through competition. Probiotics compete with pathogenic bacteria to gain scarce nutritional resources, receptor binding sites, and ecological niches in the intestinal lumen, thus limiting their growth and survival (Latif et al., 2023). Some commensal bacteria produce antibacterial bacteriocins that directly inhibit pathogenic bacteria; for example, nisin G suppresses *Fusobacterium nucleatum*, which is linked to colorectal cancer, in the *in vitro* colonic environment, but it has minimal impact on normal bacteria (Figure 1B; Lawrence et al., 2025).

The host regulates the gut microbiota by secreting antimicrobial molecules. Angiogenin (ANG), an endogenous antimicrobial protein, maintains gut microbiota diversity and homeostasis. Lack of ANG leads to overgrowth of α-Proteobacteria, decreased butyrate levels, and increased pro-inflammatory cytokines, which may cause intestinal inflammation (Figure 1B; Sun et al., 2021). Other antimicrobial proteins serve dual functions, such as human intestinal α-defensin 5 (HD5), which not only exhibits antibacterial activity but also binds to the P2Y11 receptor on the colonic epithelium and increases *Shigella*’s adhesion and invasion capabilities (Xu et al., 2025). This complexity in host, microbiota, and pathogen interactions warrants careful evaluation of antimicrobial peptide-based intervention strategies.

#### 2.4.3 Gut microbiota and immune diseases

The absence or imbalance of gut microbiota is strongly linked to various immune-related diseases in hosts. The germ-free (GF) mouse model illustrates that the microbiota play a significant role in immune development. In fact, GF mice suffer from a poorly developed mucosal immune system due to a lack of microbial stimulation. Low IgA levels are associated with impaired immune function (Shen J. et al., 2025), but colonization with gut microbiota improves serum IgA and mucosal IgA levels and partially restores mucosal immune homeostasis (Yu et al., 2021).

Gut microbiota dysbiosis plays a role in immune diseases. Gut microbiota from Crohn’s disease (CD) patients trigger spontaneous colonic inflammation in GF mice and promote the growth of opportunistic pathogens, replicating the inflammatory phenotype of CD (Sheikh et al., 2024). However, since GF mice have developmentally imperfect immune systems, it remains unclear whether the inflammatory response after transplantation is caused by the pathogenic effects of the transplanted microbiota or by an already abnormal immune background. Therefore, this study can indirectly reveal that gut microbiota dysbiosis occurring in the immature stage of the individual immune system may be one of the key factors in the development of CD. To elucidate a srtonger causal relationship between disease onset and gut microbiota dysbiosis in immunocompetent individuals, it will be necessary to develop more complex animal models and integrate multi-omics approaches to systematically decipher the specific mechanisms underlying this association. This will serve as a critical foundation for advancing the development of immune-modulating therapies targeting the gut microbiome.

### 2.5 Gut microbiota, neurobehavior, and brain health

The gut microbiota establishes a complex, bidirectional communication network with the host nervous system via the GBA. Its metabolites can traverse tissue barriers and, by integrating neural, immune, and endocrine signals, significantly affect the host’s neural behavior, brain function, and the progression of neurodegenerative diseases.

Gut microbiota metabolites are essential for maintaining neural structure and function. SCFAs can cross the blood-brain barrier through monocarboxylate transporters (MCTs), promote brain-derived neurotrophic factor (BDNF) expression, activate the downstream Phosphatidylinositol3-kinase (PI3K)/Protein kinase B (Akt) pathway, increase neuronal survival, improve synaptic plasticity, and provide cognitive enhancement and neuroprotection (Figure 1C; Facchin et al., 2024; Liu et al., 2025). Microbiota metabolites are also critical environmental factors for microglia, maintaining homeostasis and proper immune function. SCFAs support the maturation and normal morphology of microglia under physiological conditions, and N6-carboxymethyllysine (CML) can trigger oxidative stress responses and mitochondrial damage in microglia (Schaible et al., 2025). This suggests that gut-derived metabolites have a bidirectional regulatory effect on brain health.

Second, gut microbiota can indirectly affect neural behavior through endocrine-neural linkages. Microbial compounds like SCFAs, BAs, and 3-indoxyl sulfate bind to enteroendocrine cells, activating the gut-vagal nerve axis, which regulates the excitability of central nervous system neurons, thereby affecting appetite, stress response, and mood (Jameson et al., 2025). The gut microbiota also synthesizes neurotransmitters such as serotonin, dopamine, norepinephrine, and γ-aminobutyric acid, activating the myenteric and submucosal plexuses of the enteric nervous system (Figure 1C; Hamamah et al., 2022).

Dysbiosis plays a role in abnormal neurobehavior and neurodevelopmental disorders. GF mice are aggressive, but early life colonization can reverse this behavior (Watanabe et al., 2021). This demonstrates the role of the microbiota in early neurobehavioral development. Further studies show that supplementation with *Lactobacillus reuteri* improves social deficits in mouse models of neurodevelopmental disorders, with the effective window extending into adulthood (Buffington et al., 2021). This suggests that microbiota intervention is specific to certain age stages and emphasizes the importance of the “critical developmental time window” in microbiota-behavior interactions.

Gut microbiota dysbiosis is considered a potential peripheral factor in Alzheimer’s disease (AD). Studies show that microbiota imbalance can lead to abnormal gene expression and altered microglial subtypes similar to those found in AD brains (Huang et al., 2023). Furthermore, transplanting microbiota from AD patients into healthy mice can induce AD-like neuropathological changes (Upadhyay et al., 2025). Intervention strategies targeting the microbiota can also be neuroprotective. For example, supplementation with *Bacteroides ovatus* or its metabolite lysophosphatidylcholine (LPC) significantly improves AD-related pathological indicators by inhibiting ferroptosis and reducing the overactivation of microglia and astrocytes (Zha et al., 2025). This provides theoretical support for microbiota- or metabolite-based interventions in neurodegenerative diseases.

The gut microbiota is important for neural development, neurophysiology regulation, and the progression of neurodegenerative diseases. Future regulatory approaches targeting the gut microbiota and its metabolites will create a new paradigm for treating neurodegenerative diseases, promising new approaches for abnormal neural behavior and degenerative diseases.

### 2.6 Host-driven regulatory mechanisms shaping the gut microbiota

The host is not only passively subject to colonization but also actively shapes and regulates the composition and metabolic activity of the gut microbiota through many layers of interrelated regulatory mechanisms. These host-induced changes are mediated by food, mucosal immune responses, endogenous metabolic products, the physicochemical features of the intestinal environment, and lifestyle factors. Together, they create a bidirectional regulatory system that maintains microbial homeostasis and supports overall host health.

#### 2.6.1 Effects of the host’s internal environment on the gut microbiota

The intestine, as the ecological niche for the microbiota, exhibits segmental differences in structure and function that significantly drive the colonization and succession of microbial communities. From the stomach to the colon and rectum, the digestive tract undergoes notable gradient changes in pH, oxygen, nutrients, and fluid dynamics. Together, these factors define the regional features of the microbiota.

The pH value of the intestinal lumen gradually increases from the proximal to the distal small intestine. In the proximal colon, the production of SCFAs increases as dietary fiber undergoes fermentation by gut bacteria, leading to a further decrease in lumenal pH at this location (Yamamura et al., 2023). The pH levels can influence the structure and function of the gut microbiota by regulating enzyme activity. The gut microbiota is sensitive to changes in intestinal pH, and both excessively high and excessively low pH levels are detrimental to most gut microorganisms. Therefore, modifying the intestinal luminal pH can theoretically alter microbiota. Studies suggest that increasing the pH in the environment can enhance the richness of the luminal content community and increase overall SCFAs levels, but decrease the evenness of mucosal microbiota (Firrman et al., 2022). Numerous studies have now identified associations between alterations in intestinal pH and gut diseases such as IBD and colorectal cancer. This may involve the selective influence of intestinal pH on the structure and function of gut microbiota.

Second, the intestine has characteristic oxygen gradients: the longitudinal gradient shows higher oxygen levels in the proximal part compared to the distal part, and the transverse gradient shows sharp drops from mucosa to lumen (Konjar et al., 2021). Facultative anaerobes consume oxygen to maintain a local hypoxic niche in the intestine, regulating the intestinal oxygen balance and promoting the establishment of anaerobic bacteria. Disruption of this oxygen balance can lead to alterations in the microecological structure and damage to the mucus barrier, thereby worsening intestinal inflammation (Konjar et al., 2021; Zong et al., 2024).

Nutritional substrates act as selective pressures that directly shape the competitive environment of the microbiota. Undigested food, digestive enzymes, bile acids, mucins, and host secretions in the intestinal lumen provide essential resources for microbiota survival. During fasting, the reduced supply of substrates paradoxically increases microbiota diversity. In particular, the number of butyrate-producing and mucin-degrading bacteria, such as *Akkermansia, Parabacteroides*, and *Muribaculum*, rises, while *Lactobacillus* and *Bifidobacterium* decrease. When the normal diet is resumed, the microbiota structure changes reversibly (Zhang et al., 2023). This shows that the microbiota is robust within a steady-state range and that microecological interventions may be feasible and potentially reversible.

The host’s neuroendocrine system controls the microbiota structure by regulating the intestinal environment. For example, leptin, a metabolic signaling factor, can rapidly and transiently alter the microbiota structure in certain intestinal regions and affect metabolic pathways such as amino acids and vitamins. This may involve changes in intestinal peristalsis and remodeling of the ganglion structure (Toledo et al., 2025). In summary, the structure of the gut microbiota is regulated by multiple factors, including the mammalian immune system, endocrine pathways, and neural pathways, thereby enabling the host to adapt to various physiological conditions.

#### 2.6.2 Dynamic regulation of the gut microbiota by the host under different physiological states

The host’s physiological state continuously influences the gut microbiota through dynamic, multi-level mechanisms, serving as a core source of microbiota differences among individuals.

Aging affects the composition of the microbiota. Older adults have decreased diversity in gut microbiota and a decline in the population of probiotics, such as *Bifidobacterium* (Nagpal et al., 2018; Xiao et al., 2021). Age-related factors include slower intestinal peristalsis, reduced mucosal barrier function, weakened immune responses, and changes in mucus production, all of which hinder stable colonization and metabolic activity of the microbiota (Nagpal et al., 2018). Genome-wide association studies also suggest that single nucleotide polymorphism sites in the arginine synthesis pathway of *Bifidobacterium longum* are linked to host age, although longitudinal studies are needed (Xiao et al., 2021).

Second, the host’s gender affects microbiota composition through the action of sex hormones. These hormones regulate intestinal barrier integrity, peristaltic rhythm, and the immune system. Consequently, this regulation leads to variations in microbiota diversity, the proportion of commensal bacteria, and the abundance of potential pathogens among individuals of different genders (Valeri and Endres, 2021).

The host’s health plays a key role in how the microbial community is formed. For example, during the progression from subjective cognitive impairment to AD, disease severity is broadly positively correlated with gut microbiota dysbiosis. This manifests as a gradual reduction in beneficial bacteria, a relative increase in potentially pathogenic bacteria, and a tendency toward disruption in the microbial ecological network structure (Son et al., 2025). Similarly, this association has been observed in metabolic disorders. The gut microbiota exhibits significant covariation with impaired glucose tolerance, impaired fasting glucose, and type 2 diabetes mellitus (T2DM), meaning that the overall gut microbiota composition changes in response to fluctuations in blood glucose levels (Wu et al., 2020). These findings provide compelling evidence for the association between host health status and the gut microbiota, revealing a close relationship between alterations in metabolic and cognitive functions and gut dysbiosis. However, while existing studies have demonstrated correlations between the two, most are limited to cross-sectional research. To further validate the causal nature of this association, longitudinal studies across diverse populations are needed.

In summary, host physiological parameters dynamically regulate the gut microbiota through systems such as metabolism, immunity, and the neuroendocrine system, leading to microecological profiles that vary with age, gender, and health status. This plasticity underlies individual microbiota heterogeneity and offers a theoretical foundation for precise microecological interventions.

## 3 Association between gut microbiota dysbiosis and diseases

If intestinal microbiota homeostasis is disturbed (dysbiosis), the host’s health suffers in a number of ways. Dysbiosis alters the metabolism and distribution of essential compounds, impedes intestinal barrier function, and triggers abnormal immune and inflammatory responses, playing a crucial role in the onset and progression of numerous diseases. In this section, we will discuss the mechanisms and potential impacts of microbiota metabolic disorders, barrier impairment, and abnormal immune inflammation in the context of different diseases.

### 3.1 Diseases associated with imbalance of metabolic homeostasis and gut microbiota

#### 3.1.1 Type 2 diabetes mellitus

The relationship between intestinal microbiota dysbiosis and the onset and progression of T2DM is increasingly recognized. Dysbiosis can lead to altered gut permeability, facilitating the translocation of lipopolysaccharides (LPS) into the bloodstream. Increased LPS can lead to chronic low-grade inflammation, which leads to insulin resistance. Dysbiosis can also affect the production of SCFAs, which are essential for gut health and metabolism. Low production of SCFAs can affect glucose metabolism and energy homeostasis, exacerbating T2DM symptoms. Understanding these mechanisms will help develop targeted therapies aimed at the gut microbiota in T2DM (Chong et al., 2024).

**FIGURE 2:**
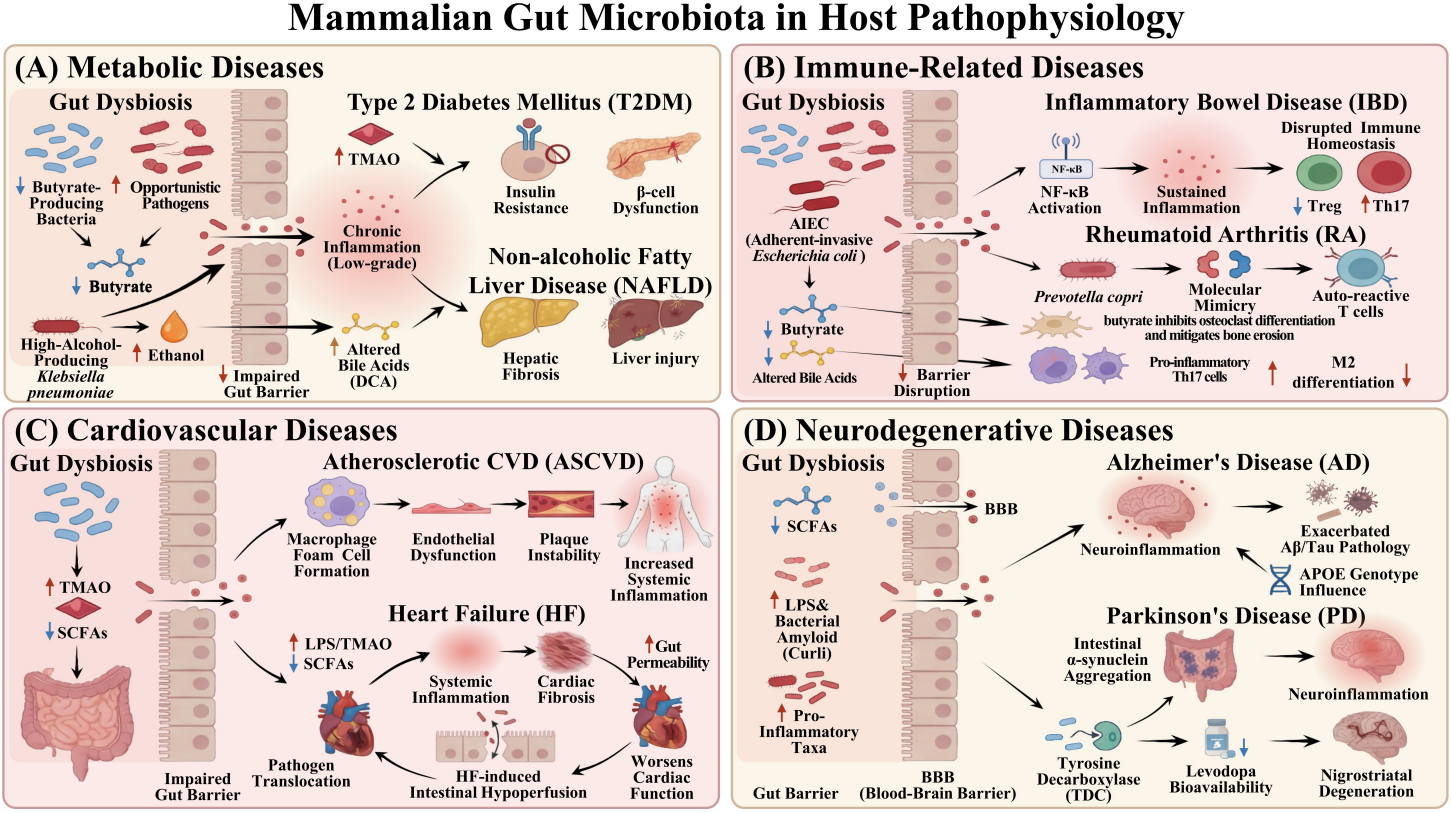
Mammalian gut microbiota in host pathophysiology. **(A)** Gut microbiota dysbiosis can alter the composition of the metabolites and disrupt intestinal barrier function, leading to chronic low-grade inflammation in the body and hence increasing the progression of metabolic diseases. **(B)** In inflammatory bowel disease dysbiosis of the gut microbiota and its metabolic products can affect intestinal mucosal barrier function, drive chronic inflammation, and breach immune tolerance. In rheumatoid arthritis, dysbiosis of the gut microbiota and its metabolic products can promote disease progression by causing immune cross-reactivity through molecular mimicry, disrupting intestinal barrier function, and compromising immune homeostasis. **(C)** In atherosclerotic cardiovascular disease, the microbial metabolite TMAO can increase risk of acute cardiovascular events by promoting macrophage foaming, disrupting endothelial function, and destabilizing plaques. In heart failure, dysregulation of the gut microbiota and its metabolites, coupled with altered intestinal hemodynamics, accelerates disease progression. **(D)** Dysbiosis of the gut microbiota can mediate enhanced neuroinflammatory responses and pathological cascade progression. Notably, specific microbial groups enriched in the gut microbiota of Parkinson’s disease patients can significantly reduce bioavailability of levodopa by degrading it. TMAO, Trimethylamine N-oxide; SCFAs, short-chain fatty acids; Th17, Helper T Cells 17; Tregs, Regulatory T Cells; LPS, Lipopolysaccharide.

The gut microbiota is considered an “endocrine organ” controlling host metabolism, and disruptions in its composition and metabolic function play a key role in T2DM. The gut microbiota structure differs from that of healthy individuals, and the abundance of Bacteroidetes generally decreases (Chong et al., 2024). Metagenomic association analysis further shows a decrease in butyrate-producing bacteria, such as *Roseburia* and *Faecalibacterium*, in T2DM patients, and an increase in opportunistic pathogens like *Escherichia-Shigella*. Functional anomalies are also observed, including the weakening of the SCFAs pathway. These changes form the core pathological basis of T2DM: endotoxemia, chronic low-grade inflammation, and insulin resistance (Figure 2A; Chong et al., 2024; Singh A. et al., 2025).

A reduction in butyrate can compromise the energy supply to intestinal epithelial cells and decrease tight junction protein expression (Chong et al., 2024; Krauze et al., 2025). This compromise allows pathogen-associated molecular patterns (PAMPs), such as LPS, to enter the circulation and activate the Toll-Like Receptor 4-Nuclear Factor Kappa-Light-Chain-Enhancer of Activated B Cells (TLR4-NF-κB) signaling pathway, which releases pro-inflammatory cytokines such as tumor necrosis factor-alpha (TNF-α) and interleukin-6 (IL-6), thereby triggering chronic low-grade inflammation. It also worsens insulin resistance by disrupting glucose uptake and insulin signaling, and increasing oxidative stress (Chong et al., 2024; Krauze et al., 2025). While butyrate inhibits the LPS-TLR4 pathway and exhibits anti-inflammatory activity at certain concentrations, it is often insufficient during dysbiosis and provides only limited anti-inflammatory effects (Luo et al., 2021; Krauze et al., 2025).

Furthermore, the gut microbiota metabolite trimethylamine N-oxide (TMAO), at concentrations associated with T2DM, can disrupt calcium homeostasis and induce endoplasmic reticulum stress. This disruption directly impairs the function of pancreatic islet β cells and promotes their apoptosis, actively advancing the progression of the disease (Kong et al., 2024).

There is a bidirectional correlation between the gut microbiota and the host’s metabolic state. The gut microbiota exhibits different structures and functions under different hyperglycemic states, and these differences are closely related to insulin resistance (Wu et al., 2020). This indicates that targeting the gut microbiota could lead to an alternative strategy for preventing or delaying T2DM. However, most existing studies employ cross-sectional designs, and the causal relationship requires further verification through longitudinal cohort studies.

#### 3.1.2 Non-alcoholic fatty liver disease

Gut microbiota dysbiosis is considered one of the major causes of non-alcoholic fatty liver disease (NAFLD). In patients with NAFLD, gut microbiota diversity decreases significantly; specifically, Bacteroidetes and Proteobacteria are more abundant, while Firmicutes are less abundant (Xia et al., 2022). At the genus level, the gut bacteria *Escherichia*, *Prevotella*, and *Streptococcus* are more abundant, whereas beneficial bacteria such as *Coprococcus*, *Faecalibacterium*, and *Ruminococcus* are less abundant (Li et al., 2021). Low-diversity, pro-inflammatory microbiota structures contribute to liver lipid metabolism disorders, inflammation, and fibrosis through various mechanisms (Figure 2A).

Gut microbiota dysbiosis can compromise intestinal barrier function (Liu et al., 2023) and allow LPS to escape into portal vein circulation. Once in circulation, LPS activates the NLRP3 inflammasome in the liver, and Kupffer cells and liver parenchymal cells release pro-inflammatory molecules. These mediators attack liver cells directly and also stimulate collagen formation and fibrosis by activating hepatic stellate cells (Seo H. Y. et al., 2023). This provides evidence of the importance of immune imbalance in the gut-liver axis during the progression of NAFLD.

Further, certain metabolites from the gut can cause liver disease directly or indirectly. For example, the carriage rate of High-Alcohol-Producing *Klebsiella pneumoniae* increases in NAFLD patients. The ethanol produced enters the bloodstream and causes liver mitochondrial dysfunction, oxidative stress, and lipid accumulation, supporting the “endogenous alcohol” hypothesis (Yuan et al., 2019). Additionally, bile acid metabolism disorders are also a pathogenic pathway for NAFLD. In the intestine, primary bile acids are converted into DCA and LCA by symbiotic bacteria such as *Bacteroides* and *Clostridium* (Cai et al., 2022). Dysbiosis can lead to increased DCA levels, which are not only directly toxic but also inhibit FXR signaling pathways in the liver and intestine, promoting lipid accumulation (Jiao et al., 2018; Cai et al., 2022).

The gut microbiota interacts with the host via various mechanisms, including barrier leakage, immune imbalance, and metabolic toxins, which contribute to the onset and progression of NAFLD. These interactions provide a theoretical foundation for gut microbiota-targeted intervention strategies, such as dietary changes, probiotics, and prebiotics.

### 3.2 Inflammatory and autoimmune diseases

#### 3.2.1 Inflammatory bowel disease

Multi-omics studies have shown that gut microbiota dysbiosis is a key factor in IBD development. Individuals with IBD show reduced microbiota diversity, a reduction in butyrate-producing bacteria (*Faecalibacterium prausnitzii* and *Roseburia intestinalis*), an increase in pro-inflammatory bacteria (*Ruminococcus gnavus*, *Bacteroides fragilis*, and *Escherichia coli*), and functional abnormalities (enhanced LPS synthesis pathway and suppressed propionate metabolism) (Ning et al., 2023).

Gut microbiota dysbiosis accelerates the progression of IBD (Figure 2B). The lack of butyrate decreases the energy supply to the colon and weakens tight junction proteins (Mukhopadhya and Louis, 2025). Adherent-invasive *Escherichia coli* invades the intestinal epithelium and macrophages, activates the NF-κB pathway, and triggers the release of pro-inflammatory molecules such as TNF-α and IL-6. It also upregulates myosin light chain kinase (MLCK), internalizes tight junction proteins, and creates a positive “leakage-inflammation” feedback loop (Shawki and McCole, 2017). Butyrate depletion can also inhibit the differentiation and immunosuppressive function of Tregs (Recharla et al., 2023). The reduction in bacteria-derived bile acids in IBD can promote the polarization of pro-inflammatory macrophages M1 and Th17 cells and inhibit M2 macrophages and Treg differentiation, further disrupting immune tolerance (Biagioli et al., 2024).

Different strains can have different immunomodulatory effects in IBD. For example, the capsular polysaccharide A (PSA) of *Bacteroides fragilis* promotes Treg cell differentiation and the secretion of anti-inflammatory cytokines (Telesford et al., 2015), while enterotoxigenic *Bacteroides fragilis* (ETBF) secretes *Bacteroides fragilis* toxin, which worsens intestinal inflammation and potentially induce tumorigenesis (Chung et al., 2018). Thus, microbiota intervention strategies for IBD must emphasize strain-level identification and functional evaluation to ensure the safe and effective clinical applications.

#### 3.2.2 Rheumatoid arthritis

Rheumatoid arthritis (RA) is a common autoimmune disease. Multi-omics studies show that gut microbiota dysbiosis is caused by decreased α-diversity, decreased abundance of butyrate-producing bacteria, and an abnormal enrichment of pro-inflammatory bacteria such as *Streptococcus* (Wang Y. et al., 2022). Newly diagnosed RA patients have an increased abundance of *Prevotella copri* in their gut (Pianta et al., 2017a). Certain proteins belonging to this bacteria share high sequence homology with two autoantigens in synovial tissue—N-acetylglucosamine-6-sulfatase (GNS) and filamin A (FLNA), potentially triggering Th1 cell cross-reactions through molecular mimicry, which can lead to synovial damage (Pianta et al., 2017b). Long-term studies suggest that gut microbiota dysbiosis may affect intestinal barrier function before disease onset, causing immune cells to accumulate in the mucosa and migrate to the synovium, thereby remotely controlling RA (Tajik et al., 2020).

Butyrate exhibits anti-inflammatory properties and inhibits osteoclast differentiation and bone erosion (He et al., 2022). Gut microbiota dysbiosis depletes butyrate and can accelerate the pathological progression of RA through various pathways. Bile acid metabolic disorders play a role in immune regulation. In RA patients, the abundance of *Parabacteroides distasonis* is notably reduced. Supplementation with *Parabacteroides distasonis* restores certain secondary bile acids, inhibits Th17 differentiation, promotes M2 macrophage polarization, and alleviates joint inflammation (Sun et al., 2023). Gut microbiota dysbiosis accelerates the pathological progression of RA through networks involving abnormal immune regulation, barrier disruption, and metabolic disorders, which offer theoretical foundations for interventions aimed at restoring microbiota balance (Figure 2B).

### 3.3 Cardiovascular diseases

Cardiovascular disease (CVD) is one of the leading causes of death worldwide. Atherosclerotic cardiovascular disease (ASCVD) is a primary type of CVD and is influenced not only by dyslipidemia. One manifestation of ASCVD is gut microbiota dysbiosis, with more pathogenic bacteria such as *Megamonas*, *Veillonella*, and *Streptococcus*, and fewer anti-inflammatory bacteria such as *Bifidobacterium* and *Roseburia* (Alexandrescu et al., 2024). Pro-inflammatory bacteria similar to those present in gut microbiota are also found in plaques, suggesting that microbiota translocation may be involved in local inflammation (Pisano et al., 2023).

TMAO, a crucial metabolite derived from gut microbiota, plays a significant role in promoting atherosclerosis (Figure 2C). It promotes the expression of inflammatory including cytokines TNF-α and IL-6, expression and regulates macrophage foaming through the CD36/MAPK/JNK pathway. TMAO also disrupts plaque stability by causing endothelial pyroptosis, inhibiting antioxidant pathways, and stimulating thrombus formation (Geng et al., 2018; Zhai et al., 2025). A lack of SCFAs also increases systemic inflammation and promotes plaque formation (Brandsma et al., 2019). A high-fat diet or irregular daily routine can disrupt the circadian rhythm of gut microbiota, which leads to increased TMAO levels and lower SCFAs levels. This increases the risk of early morning vascular endothelial dysfunction and thrombosis (Zhai et al., 2025).

In heart failure (HF), gut microbiota dysbiosis exhibits a “double-hit” pattern (Figure 2C). First, the population of beneficial bacteria declines, while pro-inflammatory bacteria proliferate. This shift is accompanied by increased production of LPS and TMAO, along with reduced synthesis of SCFAs (Cui et al., 2018). TMAO can promote the transformation of cardiac fibroblasts into myofibroblasts and aggravate cardiac fibrosis and cardiac functional injury (Yang et al., 2019). Second, decreased cardiac output results in insufficient intestinal perfusion and venous congestion, which heighten intestinal permeability (Mahenthiran et al., 2024). This facilitates the translocation of LPS into the bloodstream, activating the TLR4/NF-κB inflammatory pathway and worsening myocardial injury (Zhang et al., 2025). Additionally, the reduction in SCFAs diminishes their anti-inflammatory, energy-providing, and microenvironment-repairing functions (Palm et al., 2022). Gut microbiota and its metabolites play a role throughout the progression from ASCVD to HF through translocation, metabolism, and microenvironment reconstruction, providing a new avenue for precise intervention.

### 3.4 Neurodegenerative diseases

#### 3.4.1 Alzheimer’s disease

AD is characterized by β-amyloid (Aβ) deposition and excessive tau protein phosphorylation. Changes in gut microbiota can occur before cognitive impairment and continue throughout AD. As the disease progresses, the microbiota decreases its α-diversity, weakens its microbial network, increases potentially pathogenic bacteria, and gradually decreases butyrate-producing bacteria (Son et al., 2025).

Dysbiosis plays a role in AD pathology (Figure 2D). Bacterial amyloid curli can activate the neuroendocrine-vagus nerve axis, which increases Aβ deposition and neuroinflammation (Das et al., 2022; Sánchez-Tapia et al., 2023). A lack of butyrate and high levels of LPS increase endothelial dysfunction and systemic inflammation (Marizzoni et al., 2020). A deficiency of SCFAs and an abnormal transcriptome can affect the expression of genes associated with synaptic plasticity and aging (Hu et al., 2025). Individuals carrying the apolipoprotein E4 (*APOE4*) allele exhibit reduced levels of butyrate-producing bacteria and SCFAs in their gut microbiome, suggesting that the gut microbiota may act as an intermediary mediating the influence of the APOE gene on AD risk (Tran et al., 2019). Depletion of gut microbiota by antibiotics can improve brain atrophy, tau pathology, and abnormal glial cell activation in AD model mice (Seo D. O. et al., 2023).

#### 3.4.2 Parkinson’s disease

Parkinson’s disease (PD), the second most common neurodegenerative disorder after AD, is characterized by the progressive loss of dopaminergic neurons in the substantia nigra pars compacta and misfolding and aggregation of α-synuclein (α-syn). Recent studies have shown that individuals with PD exhibit structural and functional disturbances in the gut microbiota. At the taxonomic level, PD-associated dysbiosis is characterized by a decrease in SCFA-producing bacteria and an overrepresentation of lactic acid-producing bacteria. Functionally, this imbalance is represented by impaired SCFA biosynthesis and enhanced microbial pathways involved in the degradation of exogenous compounds (Romano et al., 2025).

Gut microbiota dysbiosis contributes to PD progression through immune dysregulation, disruption of the intestinal barrier, and altered metabolic profiles (Figure 2D). Specifically, dysbiosis in PD disrupts mucosal immune homeostasis, promotes systemic inflammation, increases intestinal permeability, and facilitates the abnormal aggregation and phosphorylation of α-syn, eventually enhancing α-syn propagation from gut to brain and dopaminergic neuronal degeneration (Munoz-Pinto et al., 2024). A lack of SCFAs can increase barrier permeability, activate microglia, inhibit anti-inflammatory pathways, and accelerate the aggregation and intracerebral spread of α-syn (Ravi et al., 2025). Curli produced by gut bacteria can accelerate pathogenic aggregation of α-syn, with the vagus nerve acting as a potential pathway for its transmission to the central nervous system (Sampson et al., 2020; Han et al., 2025). Clinical results show that truncal vagotomy may reduce the risk of PD (Liu et al., 2017).

In addition, some strains of lactic acid bacteria can degrade levodopa in the intestinal lumen by expressing tyrosine decarboxylase (TDC), which reduces the drug’s bioavailability (Romano et al., 2025). Controlling strains with high TDC expression or improving the intestinal environment can prevent levodopa degradation and delay the progression of PD.

## 4 Intervention strategies for gut microbiota regulation

### 4.1 Dietary intervention

Diet plays an important role in mammalian health and disease development. A balanced diet helps maintain homeostasis and can also be used to treat chronic diseases. One way diet affects mammals is through the regulation of gut microbiota.

A high fiber diet can contribute to the abundance and diversity of gut microbiota, improve microbiota structure, and increase the abundance of beneficial bacteria such as *Parabacteroides* and *Ruminococcaceae*, while reducing the presence of pro-inflammatory bacteria such as *Clostridium* (Wang Z. et al., 2022). It also provides substrates for SCFA-producing bacteria to increase SCFA levels in the body. SCFAs are important for maintaining the energy supply to the intestinal epithelium, strengthening barrier function, and controlling immune inflammation and metabolism. This mechanistic basis provides the potential for high-fiber diets to be used in the treatment of chronic diseases such as IBD, T2DM, CVD, and neurological disorders.

In contrast, a long-term high-fat diet can shift gut microbiota toward the “pro-inflammatory and pro-obesity” state (Imdad et al., 2024), with a lower Bacteroidetes-to-Firmicutes ratio, lower microbiota diversity, and higher levels of harmful bacteria. This imbalance in microbiota is considered one of the main mechanisms through which a high-fat diet promotes obesity and metabolic diseases.

Beyond the type of diet, the timing of eating, or dietary rhythm, significantly affects gut microbiota. Intermittent fasting can improve gut microbiota diversity, increase beneficial bacteria such as *Bifidobacterium* and *Lactobacillus*, and improve the host’s lipid profile (Khan et al., 2022). Intermittent fasting restores the metabolic rhythm of SCFAs by controlling gut microbiota function, and SCFAs, as important signaling molecules, act on multiple organs in the body through circulation, improving metabolic health (Ceperuelo-Mallafré et al., 2025). Feeding rhythms regulate segmented filamentous bacteria (SFB) attachment rhythms, which in turn regulate the rhythmic expression of antimicrobial proteins, thereby affecting resistance to infection (Brooks et al., 2021). However, the effects of dietary interventions depend on individual differences. Some studies show that identical dietary interventions can actually increase differences in gut microbiota among individuals (Vermeulen et al., 2025). Future precision nutrition interventions should take into account baseline microbiota, genetic background, immune status, and metabolic characteristics of the host to create customized plans for regulating gut microbiota.

### 4.2 Intervention of probiotics and prebiotics

Probiotics are living organisms that provide health benefits to the host when consumed in sufficient quantities. They improve intestinal health by strengthening the barrier, enhancing beneficial flora, increasing beneficial metabolites, improving mucosal immunity, and decreasing inflammation. They also prevent pathogenic bacterial colonization through competitive exclusion and metabolic interference, thereby maintaining intestinal homeostasis (Du et al., 2025; Qu et al., 2025). Some probiotics also activate tryptophan metabolism, upregulating indole-3-lactic acid production (Du et al., 2025), which has anti-inflammatory and promotes intestinal repair.

Traditional probiotic products typically rely on strains chosen based on empirical knowledge. These strains offer a broad array of functions but lack targeted effects, and their mechanisms of action are not fully understood, resulting in generalized efficacy issues. Recently, next-generation probiotics have emerged due to their advantages, including well-defined strain functions that allow personalized treatment of particular diseases and can be precisely engineered using multi-omics approaches (Jan et al., 2024). However, technical difficulties persist for next-generation probiotics, and their long-term safety and efficacy must be tested through randomized controlled trials.

Prebiotics are substances that the host does not digest or absorb but are selectively used by probiotics. For example, fructooligosaccharides can be consumed by probiotics to help them grow and inhibit pathogenic bacterial colonization. The SCFAs produced during the fermentation of prebiotics in the intestinal tract are essential for their health benefits (Singh P. et al., 2025). Unlike probiotics, prebiotics do not require the introduction of live bacteria, have stable physical and chemical properties, and can be easily integrated into functional foods or pharmaceuticals. However, effects vary among individuals and depend on the dose; excessive intake may cause discomfort, such as abdominal distension.

Probiotic and prebiotic interventions have shown promise for disease prevention and treatment, but their effectiveness differs according to strain types, host conditions, and disease types, leading to inconsistent reproducibility of results (Sharpton et al., 2019). In the future, a predictive model for microecological interventions could be developed using artificial intelligence to identify the best strain combinations and suitable populations from vast data sets. Large-scale clinical trials are needed to support their long-term effectiveness and safety.

### 4.3 Fecal microbiota transplantation

FMT involves transferring fecal microbiota from healthy donors into a patient’s gut to restore the balance of the recipient’s gut microbiome. By reconstructing microbiota diversity, optimizing microbiota structure, inhibiting pathogens, and regulating immune function, this method can correct gut microbiota dysbiosis and potentially treat various diseases (Brunse et al., 2019; Reddi et al., 2025).

In treating recurrent *Clostridioides difficile* infection (rCDI), FMT has shown high efficacy, with cure rates ranging from 70 to 91% in clinical studies, far exceeding those of antibiotics (Weerakoon et al., 2025). FMT could also be useful in other diseases, such as irritable bowel syndrome, metabolic disorders, and immune diseases.

However, as FMT has become more widely used in clinical practice, risks and challenges have become apparent. In 2019, two patients developed bacteremia from highly drug-resistant *Escherichia coli* within days of FMT; one died. Tracing showed that the resistant strains were from the same donor (DeFilipp et al., 2019), suggesting that FMT could serve as a conduit for the horizontal transmission of antimicrobial-resistant pathogens. Furthermore, the colonization efficiency of donor strains is low. Metagenomic tracking showed that only 10.8% of donor strains can stably colonize long-term, while most are cleared or fail to establish (Podlesny et al., 2022). Factors influencing colonization efficiency include the baseline structure of the recipient’s gut, antibiotic pre-treatment, donor-recipient strain compatibility, and individual strain ecological adaptability.

FMT provides a direct and effective approach to gut microbial reconstruction and can be used to treat refractory dysbiosis diseases. However, balancing therapeutic efficacy and safety remains a priority for future research and clinical applications. Advances in understanding colonization mechanisms, artificial intelligence-based donor–recipient matching algorithms, and personalized synthetic microbial consortia can improve the success rate and safety of FMT, resulting in better microbiome restoration.

### 4.4 Drugs and antibiotics

Drug interactions with the gut microbiota are bidirectional. Antibiotics, as core anti-infective agents, profoundly impact gut microbial communities. Broad-spectrum antibiotics target pathogenic bacteria but also disrupt commensal populations, leading to lower diversity, structural imbalance, and enrichment of resistance genes, resulting in dysbiosis (Suvvari et al., 2025). Epidemiological studies have linked excessive antibiotic use to an increased risk of asthma, obesity, diabetes, and other immune-metabolic disorders, with dysbiosis considered a potential mediating mechanism (McDonnell et al., 2021). Moreover, alterations in microbial composition and metabolic function may persist for months after antibiotic cessation—a phenomenon termed the “antibiotic scar” by Anthony et al. (2022) Anthony et al. (2022). This long-term and potentially irreversible effect underscores the importance of rational antibiotic use, awareness of long-term risks, and precise microbiota-targeted interventions.

On the other hand, certain pharmaceuticals require biotransformation by the gut microbiota to generate their active forms, thereby exerting therapeutic effects. A prime example of this is sulfasalazine, which is cleaved by azoreductase enzymes in gut microbes, releasing sulfapyridine and 5-aminosalicylic acid (Doestzada et al., 2018). These metabolites exert the anti-inflammatory effects in the colon, playing a crucial role in the treatment of ulcerative colitis. However, the gut microbiota can also influence drug efficacy and toxicity. For example, microbial β-lactamases can hydrolyze antibiotics and protect both the host and the bacteria from antimicrobial effects, while simultaneously reducing efficacy against infections (Jeong et al., 2024). These findings prompt us to consider whether enhancing drug efficacy could be achieved by modulating the activity of relevant enzymes within the gut microbiota.

Targeting key microbial enzymes with small molecule inhibitors has been proposed as a novel tool for precise microbiota intervention. Unlike antibiotics, such approaches modulate microbial metabolism without killing bacteria, theoretically conserving beneficial functions. Moreover, 3,3-dimethyl-1-butanol (DMB) inhibits gut microbial trimethylamine (TMA) lyase, thus reducing the level of the pathogenic metabolite TMAO and decreasing the risk of CVDs such as atherosclerosis (Motte et al., 2025). Microbial TDC can convert levodopa into dopamine directly in the gut, thereby diminishing its therapeutic activity in PD (Romano et al., 2025). Based on this, Rekdal et al. developed selective TDC inhibitors, (S)-α-fluoromethyltyrosine (AFMT), which block microbial metabolism of levodopa *in vivo*, increasing plasma drug exposure and bioavailability (Maini Rekdal et al., 2019).

However, targeting microbial enzymes is a double-edged sword. Long-term use of inhibitors can induce compensatory changes in microbial metabolic networks, leading to off-target effects or resistance in strains. Most current studies are limited to animal models. Safety and efficacy evaluations require long-term studies in animals and clinical trials.

## 5 Research methods and technological advancements

### 5.1 Multi-omics integrative analysis

Metagenomics is one of the key areas of microbiome research and allows for the analysis of the gene composition and functional potential of bacteria in samples through high-throughput sequencing. This technology not only detects species composition but also predicts metabolic pathways, antibiotic resistance genes, and other functions. With the rapid development of metagenomics next-generation sequencing (mNGS), metagenomic analysis has become much faster and more accurate. For example, mNGS can measure differences in fiber-degrading genes in pigs with different physiological features. Research suggests that the gut microbiota of sows with high reproductive performance is richer in carbohydrate-active enzyme (CAZyme) genes and fiber-degrading bacteria. Fiber-degrading capability could be used as a biomarker for host health and production performance (Miura et al., 2024). However, mNGS has difficulty resolving repetitive sequences, large-scale structural variations, and strain-level resolution. Hence, hybrid sequencing approaches combine the strengths of sequencing technologies to help tackle genomes at the strain level and provide a higher resolution analytical framework for gut microbiota research (Chen L. et al., 2022).

Metagenomics can predict the potential of the microbiota but cannot directly quantify its metabolites. Metabolomics, based on the analysis of metabolites in biological samples, can indicate the metabolic activity of microbiota and the effect on the host. In gut microbiota research, targeted metabolomics can be used for the quantitative analysis of specific metabolites, such as TMAO and SCFAs. Kuo et al. (2022) found a significant correlation between serum TMAO and metabolic syndrome in patients with coronary artery disease (Kuo et al., 2022), further confirming the role of microbiota metabolites in the pathogenesis of CVDs. Multi-omics integrated analysis, combining metagenomics and metabolomics, can establish association networks from microbiota genes to metabolic functions. For example, in vascular calcification research, metagenomics identifies microbiota gene markers and metabolic pathways associated with the disease, while metabolomics validates the effects of relevant metabolites, such as methionine, on host function and correlates with disease severity (Huang et al., 2026). A dual-omics collaborative approach forms a complete evidence chain from prediction to intervention and provides a new model for understanding gut microbiota action.

Single-cell sequencing (scSeq) has moved microbiota research from a community-wide perspective to single-cell analysis. It addresses the heterogeneity of host cells, captures gene expression data from both host cells and microbiota, and shows the host-microbiota interaction network (Chen et al., 2017). Single-cell correlation analysis shows that gut microbiota can work together with immune checkpoint inhibitors to enhance antigen presentation and T-cell activation in tumors (Cao et al., 2025). Although current studies focus on host cells and rarely investigate single-cell analysis of the metabolic state of the microbiota, single-cell and microbial single-cell sequencing technologies will allow researchers to characterize the microbiota’s functional state and its interaction network with the host at single-cell and single-bacterium levels, offering new opportunities for precise research.

### 5.2 Animal models and organoids

To establish a causal link between gut microbiota and host phenotypes, and to thoroughly examine their interaction mechanisms, a reliable experimental model system is essential. GF animal models and intestinal organoids serve as the two primary tools in this field, effectively complementing one another.

GF mice have several advantages in studying the causal effects of the microbiota on host physiology, immunity, and diseases. Mice raised in germ-free conditions exhibit developmental defects in different systems, such as structural defects in gut lymphoid tissues and underdeveloped immune cells (Shen J. et al., 2025). Colonizing GF mice with specific strains or microbiota allows for the observation of effects on host physiological or disease phenotypes, thereby establishing causality. For example, transplantation of fecal bacteria from patients into GF mice can replicate disease phenotypes, thereby demonstrating the microbiota’s pathogenic role in those diseases (Sheikh et al., 2024).

The GF animal model faces challenges such as species differences, high maintenance costs, and ethical issues, which motivate researchers to seek alternative solutions. Organoid technology, closely modeling physiological conditions, provides a controllable platform with minimal ethical concerns for studying host-microbiota interactions. In intestinal research, organoids are useful for modeling IBD, personalized drug screening and toxicity evaluation, and have wide potential for application (Fatehullah et al., 2016). Using a bacteria-organoid co-culture system, researchers found that *Bacillus subtilis* promotes the differentiation of intestinal stem cells into secretory cells, improves intestinal barrier function, and reduces infection risk (Hou et al., 2022). Organoids lack a vascular system; activation with CHIR99021, FGF4, and VEGFA may induce vascular networks in intestinal organoids (Miao et al., 2025), offering new approaches for developing mature organoid models with complete microenvironmental interactions.

### 5.3 Artificial intelligence and machine learning

High-throughput omics technologies have produced vast amounts of gut microbiota data, and artificial intelligence, particularly machine learning (ML), can be used to extract patterns from these huge datasets. ML finds patterns in training data to predict new samples. ML is promising for disease prediction, diagnosis, and personalized intervention.

ML models using gut microbiota characteristics can significantly improve disease prediction accuracy. For example, the Comprehensive Data Optimization and Risk Prediction Framework (CDORPF) can predict IBD with 90.4% accuracy and has robust and generalization capabilities (Peng et al., 2024). The framework integrates and optimizes multi-source microbiota data, showing that microbiota-based ML diagnosis provides non-invasive and highly efficient advantages.

ML, combined with deep reinforcement learning and multi-objective optimization, can quickly identify the best strain combinations for specific diseases by analyzing large data on probiotic-host interactions. This allows researchers to adjust dosages and ratios with dynamic feedback for target interventions. For example, by using high-throughput gut-on-chip and unsupervised ML, researchers can quantify the differences among different probiotics against enteritis and have identified *Bifidobacterium longum 3–14* as the most effective strain for IBD among several strains (Wu et al., 2024). Such techniques allow rapid optimization of probiotic combinations, reduce animal experiments, and speed up the development of customized microbiota interventions.

However, the application of ML still faces several critical challenges, including data bias that may lead to unreliable models or results, limited model interpretability, and obstacles to reproducibility (Zhou and Zhao, 2025). Future efforts should focus on developing novel data imputation methods and ML models to enhance data quality and model interpretability. Furthermore, as ML captures correlations rather than causation, integrating diverse omics data types could deepen our understanding of the microbiome-host health interaction. This approach holds promise for building more precise models and improving predictive capabilities, thereby opening new avenues for precision medicine and drug development.

## 6 Conclusion

Gut microbiota, a critical component of the mammalian body, plays a key role in metabolism, immune regulation, and neural function. Under physiological homeostasis, gut microbiota supports metabolic balance, immune stability, and neural function of the host by producing SCFAs, controlling immune signaling, regulating GBA signals, and engaging in other metabolic pathways. When gut microbiota is imbalanced, intestinal barrier function may be compromised, immune inflammation can be uncontrolled, and metabolic signaling can be disrupted, leading to metabolic, immune-related, cardiovascular, and neurodegenerative diseases.

In recent years, multi-omics integrated analysis, single-cell sequencing, metabolomics, and metagenomics have become unprecedented tools for studying the functional potential of gut bacteria and their effects on the host. By combining metagenomics and metabolomics, associations between microbial gene markers, metabolic functions, and host phenotypes have been established, revealing an evidence chain for microbiota mechanisms. Single-cell sequencing has also revealed interactions between the host and microorganisms at the single-cell level, suggesting new insights into microbiota mechanisms for immune regulation, inflammation, and the tumor microenvironment. GF animals and organoid systems are powerful experimental tools for investigating the causality between microbiota and the host. GF mice illustrate the influence of specific microbiota on host physiology and disease behavior. Organoid models provide ethical advantages, controllability, and enable high-throughput screening experiments, particularly co-culture experiments between bacteria and the host. They can reveal molecular mechanisms by which microbiota influence stem cell differentiation, barrier function, and immune response. Together, these tools support basic research and lay the foundation for precise interventions in the future. The use of artificial intelligence and ML has improved the clinical translation of gut microbiota research. ML models using high-throughput omics data can help predict disease and risk, as well as optimize strain combinations and doses in personalized interventions. This makes the gut microbiota a possible therapeutic target in clinical trials. Multi-omics and ML also allow researchers to detect patterns in complex data, develop hypotheses, and direct experimental validation.

Despite this progress, translating gut microbiota research into clinical interventions faces several challenges. First, individual host variability results in different outcomes for the same intervention across different populations. To identify subgroups of beneficiaries, high-quality controlled trials, multi-omics approaches, and ML models are needed. Second, long-term safety and risks to ecological homeostasis should be evaluated, especially when using exogenous microbiota interventions or live biotherapeutic products (LBPs). Ethical considerations and data security must also be considered. Regulatory and legal frameworks must be provided to protect the recipients’ vulnerabilities, privacy, and to manage microbiota characteristic information.

Overall, gut microbiota research is rapidly progressing and integrating across disciplines. From decoding the “second genome” in basic science to exploring its potential as a target for disease prevention and treatment, this work has deepened our understanding of physiological and pathological mechanisms and provided a new scientific foundation for personalized medicine, precise intervention, and global health management. With the further integration of multi-omics technologies, experimental models, and artificial intelligence, gut microbiota research will achieve a closed-loop from basic discovery to clinical application, providing systematic solutions for disease prevention, treatment, and health maintenance, and making it a key component of modern medicine.
